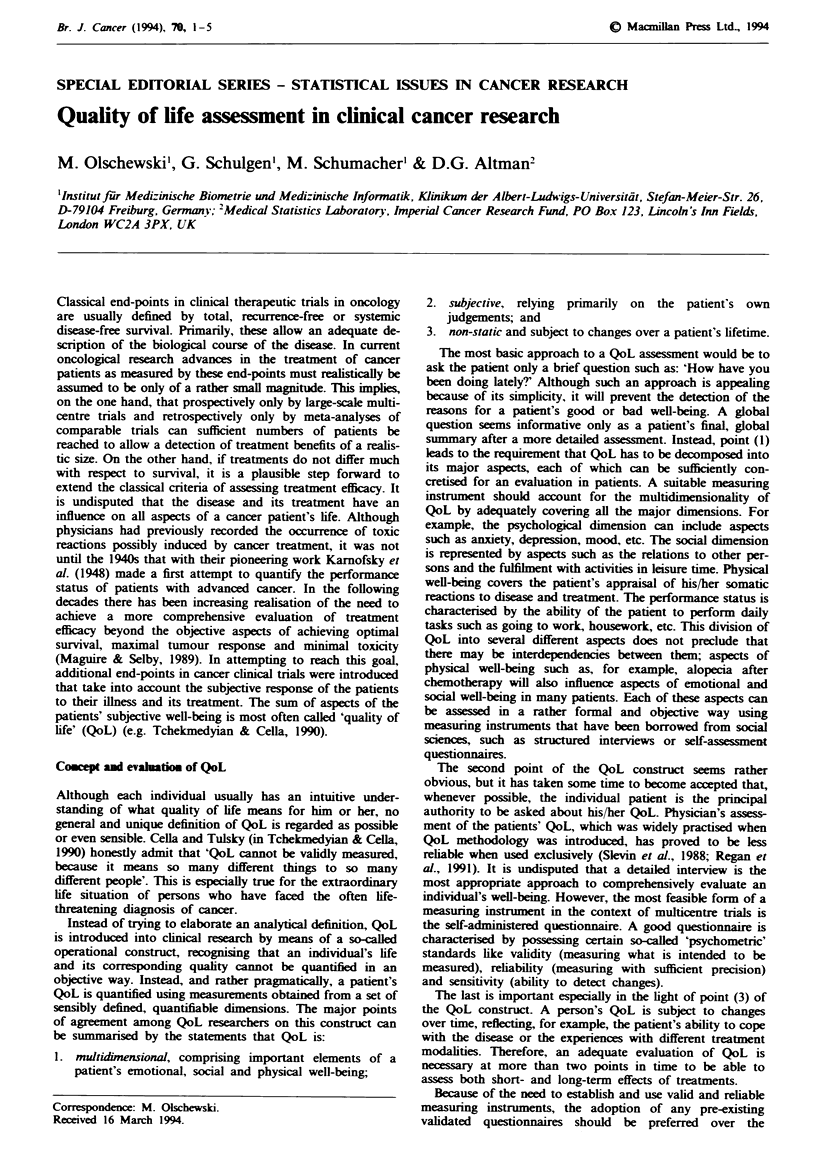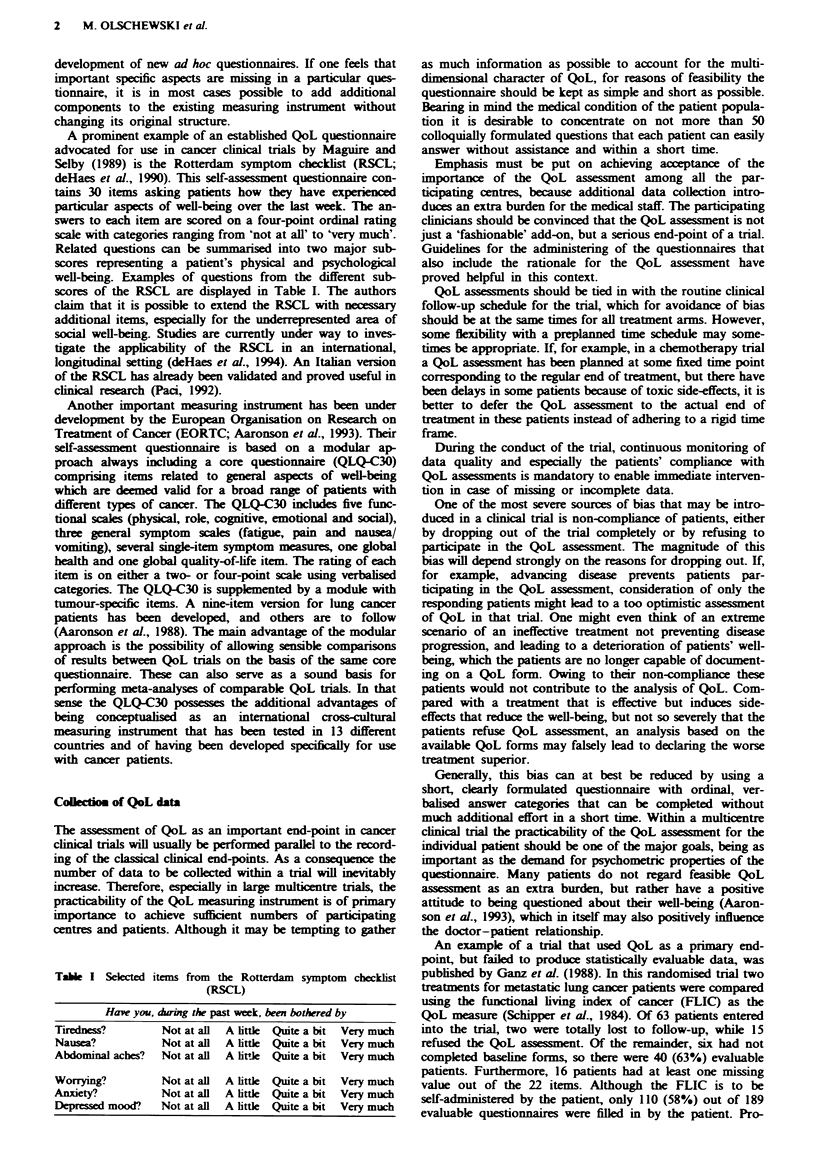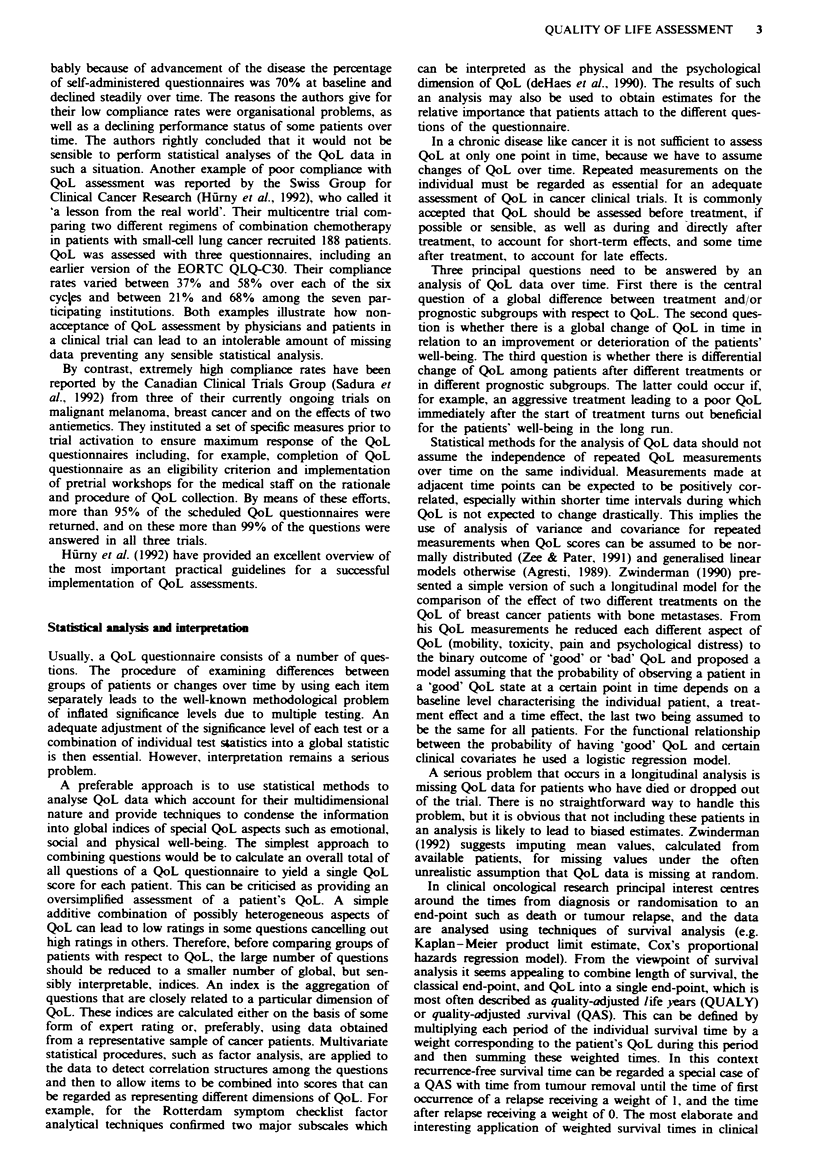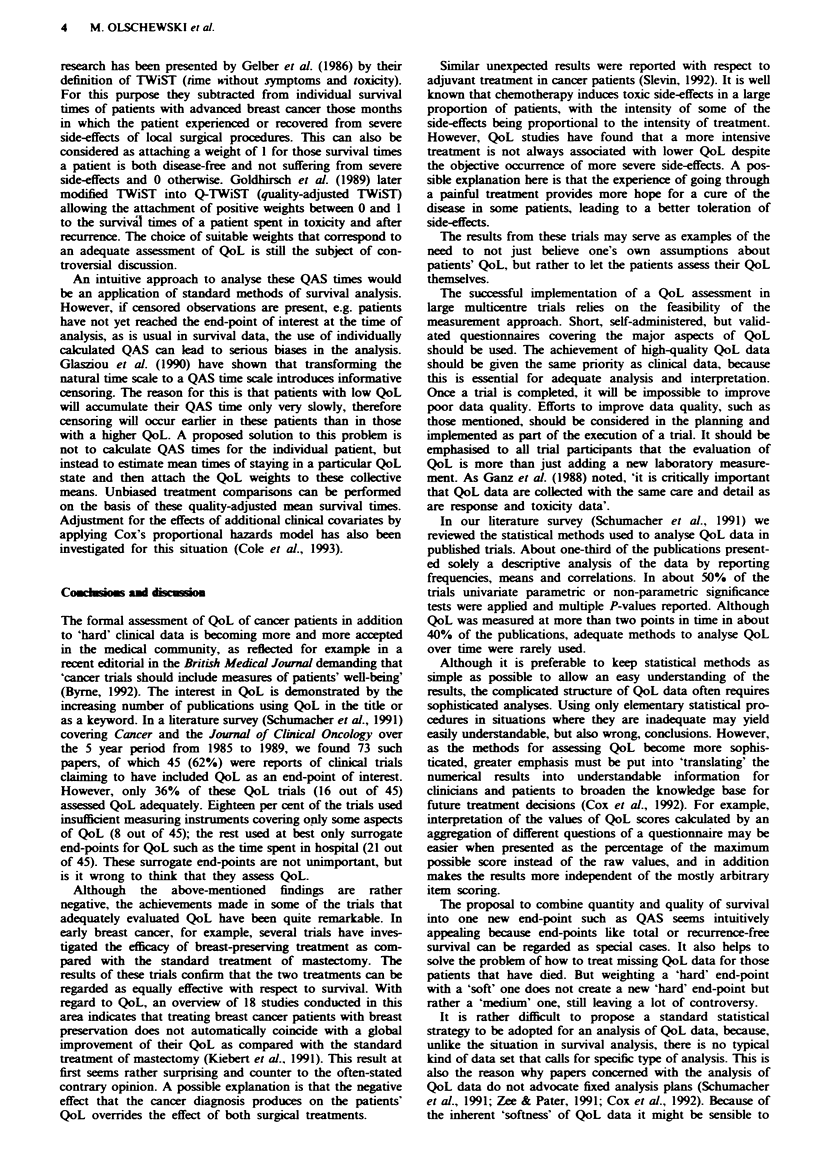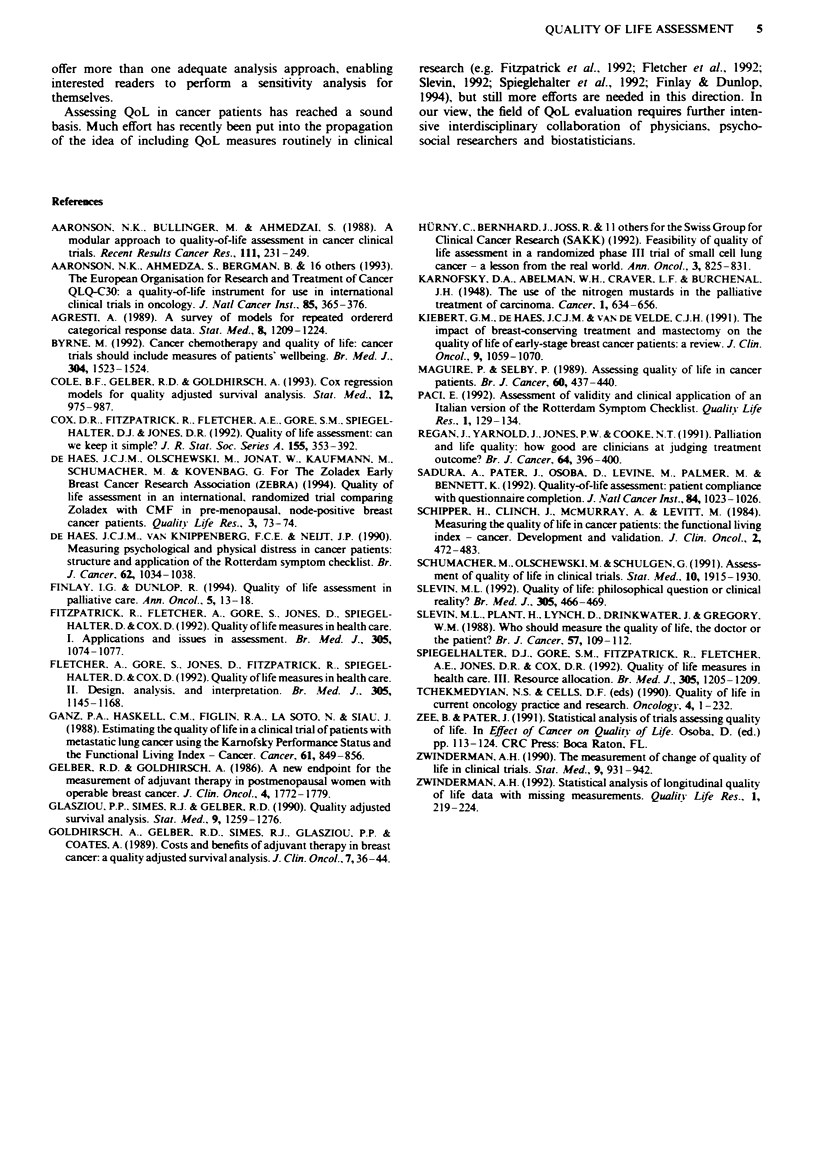# Quality of life assessment in clinical cancer research.

**DOI:** 10.1038/bjc.1994.240

**Published:** 1994-07

**Authors:** M. Olschewski, G. Schulgen, M. Schumacher, D. G. Altman


 © Macmillan Press Ltd., 1994

SPECIAL EDITORIAL SERIES - STATISTICAL ISSUES IN CANCER RESEARCH

### Quality of life assessment in clinical cancer research

M. Olschewski[1], G. Schulgen[1], M. Schumacher[1] & D.G. Altman[2]

[1]*Institut für Medizinische Biometrie und Medizinische Informatik, Klinikum der Albert-Ludwigs-Universität, Stefan-Meier-Str. 26, D-79104 Freiburg, Germany;* [2]*Medical Statistics Laboratory, Imperial Cancer Research Fund, PO Box 123, Lincoln's Inn Fields, London WC2A 3PX, UK*

Classical end-points in clinical therapeutic trials in oncology are usually defined by total, recurrence-free or systemic disease-free survival. Primarily, these allow an adequate description of the biological course of the disease. In current oncological research advances in the treatment of cancer patients as measured by these end-points must realistically be assumed to be only of a rather small magnitude. This implies, on the one hand, that prospectively only by large-scale multicentre trials and retrospectively only by meta-analyses of comparable trials can sufficient numbers of patients be reached to allow a detection of treatment benefits of a realistic size. On the other hand, if treatments do not differ much with respect to survival, it is a plausible step forward to extend the classical criteria of assessing treatment efficacy. It is undisputed that the disease and its treatment have an influence on all aspects of a cancer patient's life. Although physicians had previously recorded the occurrence of toxic reactions possibly induced by cancer treatment, it was not until the 1940s that with their pioneering work Karnofsky *et al.* (1948) made a first attempt to quantify the performance status of patients with advanced cancer. In the following decades there has been increasing realisation of the need to achieve a more comprehensive evaluation of treatment efficacy beyond the objective aspects of achieving optimal survival, maximal tumour response and minimal toxicity (Maguire & Selby, 1989). In attempting to reach this goal, additional end-points in cancer clinical trials were introduced that take into account the subjective response of the patients to their illness and its treatment. The sum of aspects of the patients' subjective well-being is most often called 'quality of life' (QoL) (e.g. Tchekmedyian & Cella, 1990).

### Concept and evaluation of QoL

Although each individual usually has an intuitive understanding of what quality of life means for him or her, no general and unique definition of QoL is regarded as possible or even sensible. Cella and Tulsky (in Tchekmedyian & Cella, 1990) honestly admit that 'QoL cannot be validly measured, because it means so many different things to so many different people'. This is especially true for the extraordinary life situation of persons who have faced the often life-threatening diagnosis of cancer.

Instead of trying to elaborate an analytical definition, QoL is introduced into clinical research by means of a so-called operational construct, recognising that an individual's life and its corresponding quality cannot be quantified in an objective way. Instead, and rather pragmatically, a patient's QoL is quantified using measurements obtained from a set of sensibly defined, quantifiable dimensions. The major points of agreement among QoL researchers on this construct can be summarised by the statements that QoL is:

1. *multidimensional*, comprising important elements of a patient's emotional, social and physical well-being;
2. *subjective*, relying primarily on the patient's own judgements; and
3. *non-static* and subject to changes over a patient's lifetime.

Correspondence: M. Olschewski.
Received 16 March 1994.

The most basic approach to a QoL assessment would be to ask the patient only a brief question such as: 'How have you been doing lately?' Although such an approach is appealing because of its simplicity, it will prevent the detection of the reasons for a patient's good or bad well-being. A global question seems informative only as a patient's final, global summary after a more detailed assessment. Instead, point (1) leads to the requirement that QoL has to be decomposed into its major aspects, each of which can be sufficiently concretised for an evaluation in patients. A suitable measuring instrument should account for the multidimensionality of QoL by adequately covering all the major dimensions. For example, the psychological dimension can include aspects such as anxiety, depression, mood, etc. The social dimension is represented by aspects such as the relations to other persons and the fulfilment with activities in leisure time. Physical well-being covers the patient's appraisal of his/her somatic reactions to disease and treatment. The performance status is characterised by the ability of the patient to perform daily tasks such as going to work, housework, etc. This division of QoL into several different aspects does not preclude that there may be interdependencies between them; aspects of physical well-being such as, for example, alopecia after chemotherapy will also influence aspects of emotional and social well-being in many patients. Each of these aspects can be assessed in a rather formal and objective way using measuring instruments that have been borrowed from social sciences, such as structured interviews or self-assessment questionnaires.

The second point of the QoL construct seems rather obvious, but it has taken some time to become accepted that, whenever possible, the individual patient is the principal authority to be asked about his/her QoL. Physician's assessment of the patients' QoL, which was widely practised when QoL methodology was introduced, has proved to be less reliable when used exclusively (Slevin *et al.*, 1988; Regan *et al.*, 1991). It is undisputed that a detailed interview is the most appropriate approach to comprehensively evaluate an individual's well-being. However, the most feasible form of a measuring instrument in the context of multicentre trials is the self-administered questionnaire. A good questionnaire is characterised by possessing certain so-called 'psychometric' standards like validity (measuring what is intended to be measured), reliability (measuring with sufficient precision) and sensitivity (ability to detect changes).

The last is important especially in the light of point (3) of the QoL construct. A person's QoL is subject to changes over time, reflecting, for example, the patient's ability to cope with the disease or the experiences with different treatment modalities. Therefore, an adequate evaluation of QoL is necessary at more than two points in time to be able to assess both short- and long-term effects of treatments.

Because of the need to establish and use valid and reliable measuring instruments, the adoption of any pre-existing validated questionnaires should be preferred over the development of new *ad hoc* questionnaires. If one feels that important specific aspects are missing in a particular questionnaire, it is in most cases possible to add additional components to the existing measuring instrument without changing its original structure.

A prominent example of an established QoL questionnaire advocated for use in cancer clinical trials by Maguire and Selby (1989) is the Rotterdam symptom checklist (RSCL; deHaes *et al.*, 1990). This self-assessment questionnaire contains 30 items asking patients how they have experienced particular aspects of well-being over the last week. The answers to each item are scored on a four-point ordinal rating scale with categories ranging from 'not at all' to 'very much'. Related questions can be summarised into two major subscores representing a patient's physical and psychological well-being. Examples of questions from the different subscores of the RSCL are displayed in Table I. The authors claim that it is possible to extend the RSCL with necessary additional items, especially for the underrepresented area of social well-being. Studies are currently under way to investigate the applicability of the RSCL in an international, longitudinal setting (deHaes *et al.*, 1994). An Italian version of the RSCL has already been validated and proved useful in clinical research (Paci, 1992).

Another important measuring instrument has been under development by the European Organisation on Research on Treatment of Cancer (EORTC; Aaronson *et al.*, 1993). Their self-assessment questionnaire is based on a modular approach always including a core questionnaire (QLQ-C30) comprising items related to general aspects of well-being which are deemed valid for a broad range of patients with different types of cancer. The QLQ-C30 includes five functional scales (physical, role, cognitive, emotional and social), three general symptom scales (fatigue, pain and nausea/vomiting), several single-item symptom measures, one global health and one global quality-of-life item. The rating of each item is on either a two- or four-point scale using verbalised categories. The QLQ-C30 is supplemented by a module with tumour-specific items. A nine-item version for lung cancer patients has been developed, and others are to follow (Aaronson *et al.*, 1988). The main advantage of the modular approach is the possibility of allowing sensible comparisons of results between QoL trials on the basis of the same core questionnaire. These can also serve as a sound basis for performing meta-analyses of comparable QoL trials. In that sense the QLQ-C30 possesses the additional advantages of being conceptualised as an international cross-cultural measuring instrument that has been tested in 13 different countries and of having been developed specifically for use with cancer patients.

### Collection of QoL data

The assessment of QoL as an important end-point in cancer clinical trials will usually be performed parallel to the recording of the classical clinical end-points. As a consequence the number of data to be collected within a trial will inevitably increase. Therefore, especially in large multicentre trials, the practicability of the QoL measuring instrument is of primary importance to achieve sufficient numbers of participating centres and patients. Although it may be tempting to gather as much information as possible to account for the multidimensional character of QoL, for reasons of feasibility the questionnaire should be kept as simple and short as possible. Bearing in mind the medical condition of the patient population it is desirable to concentrate on not more than 50 colloquially formulated questions that each patient can easily answer without assistance and within a short time.

Emphasis must be put on achieving acceptance of the importance of the QoL assessment among all the participating centres, because additional data collection introduces an extra burden for the medical staff. The participating clinicians should be convinced that the QoL assessment is not just a 'fashionable' add-on, but a serious end-point of a trial. Guidelines for the administering of the questionnaires that also include the rationale for the QoL assessment have proved helpful in this context.

QoL assessments should be tied in with the routine clinical follow-up schedule for the trial, which for avoidance of bias should be at the same times for all treatment arms. However, some flexibility with a preplanned time schedule may sometimes be appropriate. If, for example, in a chemotherapy trial a QoL assessment has been planned at some fixed time point corresponding to the regular end of treatment, but there have been delays in some patients because of toxic side-effects, it is better to defer the QoL assessment to the actual end of treatment in these patients instead of adhering to a rigid time frame.

During the conduct of the trial, continuous monitoring of data quality and especially the patients' compliance with QoL assessments is mandatory to enable immediate intervention in case of missing or incomplete data.

One of the most severe sources of bias that may be introduced in a clinical trial is non-compliance of patients, either by dropping out of the trial completely or by refusing to participate in the QoL assessment. The magnitude of this bias will depend strongly on the reasons for dropping out. If, for example, advancing disease prevents patients participating in the QoL assessment, consideration of only the responding patients might lead to a too optimistic assessment of QoL in that trial. One might even think of an extreme scenario of an ineffective treatment not preventing disease progression, and leading to a deterioration of patients' well-being, which the patients are no longer capable of documenting on a QoL form. Owing to their non-compliance these patients would not contribute to the analysis of QoL. Compared with a treatment that is effective but induces side-effects that reduce the well-being, but not so severely that the patients refuse QoL assessment, an analysis based on the available QoL forms may falsely lead to declaring the worse treatment superior.

Generally, this bias can at best be reduced by using a short, clearly formulated questionnaire with ordinal, verbalised answer categories that can be completed without much additional effort in a short time. Within a multicentre clinical trial the practicability of the QoL assessment for the individual patient should be one of the major goals, being as important as the demand for psychometric properties of the questionnaire. Many patients do not regard feasible QoL assessment as an extra burden, but rather have a positive attitude to being questioned about their well-being (Aaronson *et al.*, 1993), which in itself may also positively influence the doctor–patient relationship.

An example of a trial that used QoL as a primary end-point, but failed to produce statistically evaluable data, was published by Ganz *et al.* (1988). In this randomised trial two treatments for metastatic lung cancer patients were compared using the functional living index of cancer (FLIC) as the QoL measure (Schipper *et al.*, 1984). Of 63 patients entered into the trial, two were totally lost to follow-up, while 15 refused the QoL assessment. Of the remainder, six had not completed baseline forms, so there were 40 (63%) evaluable patients. Furthermore, 16 patients had at least one missing value out of the 22 items. Although the FLIC is to be self-administered by the patient, only 110 (58%) out of 189 evaluable questionnaires were filled in by the patient. Pro-

**Table I** Selected items from the Rotterdam symptom checklist (RSCL)

| *Have you, during the past week, been bothered by* | | | | |
|---|---|---|---|---|
| Tiredness? | Not at all | A little | Quite a bit | Very much |
| Nausea? | Not at all | A little | Quite a bit | Very much |
| Abdominal aches? | Not at all | A little | Quite a bit | Very much |
| | | | | |
| Worrying? | Not at all | A little | Quite a bit | Very much |
| Anxiety? | Not at all | A little | Quite a bit | Very much |
| Depressed mood? | Not at all | A little | Quite a bit | Very much |

bably because of advancement of the disease the percentage of self-administered questionnaires was 70% at baseline and declined steadily over time. The reasons the authors give for their low compliance rates were organisational problems, as well as a declining performance status of some patients over time. The authors rightly concluded that it would not be sensible to perform statistical analyses of the QoL data in such a situation. Another example of poor compliance with QoL assessment was reported by the Swiss Group for Clinical Cancer Research (Hürny *et al*., 1992), who called it 'a lesson from the real world'. Their multicentre trial comparing two different regimens of combination chemotherapy in patients with small-cell lung cancer recruited 188 patients. QoL was assessed with three questionnaires, including an earlier version of the EORTC QLQ-C30. Their compliance rates varied between 37% and 58% over each of the six cycles and between 21% and 68% among the seven participating institutions. Both examples illustrate how non-acceptance of QoL assessment by physicians and patients in a clinical trial can lead to an intolerable amount of missing data preventing any sensible statistical analysis.

By contrast, extremely high compliance rates have been reported by the Canadian Clinical Trials Group (Sadura *et al*., 1992) from three of their currently ongoing trials on malignant melanoma, breast cancer and on the effects of two antiemetics. They instituted a set of specific measures prior to trial activation to ensure maximum response of the QoL questionnaires including, for example, completion of QoL questionnaire as an eligibility criterion and implementation of pretrial workshops for the medical staff on the rationale and procedure of QoL collection. By means of these efforts, more than 95% of the scheduled QoL questionnaires were returned, and on these more than 99% of the questions were answered in all three trials.

Hürny *et al*. (1992) have provided an excellent overview of the most important practical guidelines for a successful implementation of QoL assessments.

**Statistical analysis and interpretation**

Usually, a QoL questionnaire consists of a number of questions. The procedure of examining differences between groups of patients or changes over time by using each item separately leads to the well-known methodological problem of inflated significance levels due to multiple testing. An adequate adjustment of the significance level of each test or a combination of individual test statistics into a global statistic is then essential. However, interpretation remains a serious problem.

A preferable approach is to use statistical methods to analyse QoL data which account for their multidimensional nature and provide techniques to condense the information into global indices of special QoL aspects such as emotional, social and physical well-being. The simplest approach to combining questions would be to calculate an overall total of all questions of a QoL questionnaire to yield a single QoL score for each patient. This can be criticised as providing an oversimplified assessment of a patient's QoL. A simple additive combination of possibly heterogeneous aspects of QoL can lead to low ratings in some questions cancelling out high ratings in others. Therefore, before comparing groups of patients with respect to QoL, the large number of questions should be reduced to a smaller number of global, but sensibly interpretable, indices. An index is the aggregation of questions that are closely related to a particular dimension of QoL. These indices are calculated either on the basis of some form of expert rating or, preferably, using data obtained from a representative sample of cancer patients. Multivariate statistical procedures, such as factor analysis, are applied to the data to detect correlation structures among the questions and then to allow items to be combined into scores that can be regarded as representing different dimensions of QoL. For example, for the Rotterdam symptom checklist factor analytical techniques confirmed two major subscales which can be interpreted as the physical and the psychological dimension of QoL (deHaes *et al*., 1990). The results of such an analysis may also be used to obtain estimates for the relative importance that patients attach to the different questions of the questionnaire.

In a chronic disease like cancer it is not sufficient to assess QoL at only one point in time, because we have to assume changes of QoL over time. Repeated measurements on the individual must be regarded as essential for an adequate assessment of QoL in cancer clinical trials. It is commonly accepted that QoL should be assessed before treatment, if possible or sensible, as well as during and directly after treatment, to account for short-term effects, and some time after treatment, to account for late effects.

Three principal questions need to be answered by an analysis of QoL data over time. First there is the central question of a global difference between treatment and/or prognostic subgroups with respect to QoL. The second question is whether there is a global change of QoL in time in relation to an improvement or deterioration of the patients' well-being. The third question is whether there is differential change of QoL among patients after different treatments or in different prognostic subgroups. The latter could occur if, for example, an aggressive treatment leading to a poor QoL immediately after the start of treatment turns out beneficial for the patients' well-being in the long run.

Statistical methods for the analysis of QoL data should not assume the independence of repeated QoL measurements over time on the same individual. Measurements made at adjacent time points can be expected to be positively correlated, especially within shorter time intervals during which QoL is not expected to change drastically. This implies the use of analysis of variance and covariance for repeated measurements when QoL scores can be assumed to be normally distributed (Zee & Pater, 1991) and generalised linear models otherwise (Agresti, 1989). Zwinderman (1990) presented a simple version of such a longitudinal model for the comparison of the effect of two different treatments on the QoL of breast cancer patients with bone metastases. From his QoL measurements he reduced each different aspect of QoL (mobility, toxicity, pain and psychological distress) to the binary outcome of 'good' or 'bad' QoL and proposed a model assuming that the probability of observing a patient in a 'good' QoL state at a certain point in time depends on a baseline level characterising the individual patient, a treatment effect and a time effect, the last two being assumed to be the same for all patients. For the functional relationship between the probability of having 'good' QoL and certain clinical covariates he used a logistic regression model.

A serious problem that occurs in a longitudinal analysis is missing QoL data for patients who have died or dropped out of the trial. There is no straightforward way to handle this problem, but it is obvious that not including these patients in an analysis is likely to lead to biased estimates. Zwinderman (1992) suggests imputing mean values, calculated from available patients, for missing values under the often unrealistic assumption that QoL data is missing at random.

In clinical oncological research principal interest centres around the times from diagnosis or randomisation to an end-point such as death or tumour relapse, and the data are analysed using techniques of survival analysis (e.g. Kaplan–Meier product limit estimate, Cox's proportional hazards regression model). From the viewpoint of survival analysis it seems appealing to combine length of survival, the classical end-point, and QoL into a single end-point, which is most often described as *q*uality-*a*djusted *l*ife *y*ears (QUALY) or *q*uality-*a*djusted *s*urvival (QAS). This can be defined by multiplying each period of the individual survival time by a weight corresponding to the patient's QoL during this period and then summing these weighted times. In this context recurrence-free survival time can be regarded a special case of a QAS with time from tumour removal until the time of first occurrence of a relapse receiving a weight of 1, and the time after relapse receiving a weight of 0. The most elaborate and interesting application of weighted survival times in clinical research has been presented by Gelber *et al.* (1986) by their definition of TWiST (*t*ime *w*ithout *s*ymptoms and *t*oxicity). For this purpose they subtracted from individual survival times of patients with advanced breast cancer those months in which the patient experienced or recovered from severe side-effects of local surgical procedures. This can also be considered as attaching a weight of 1 for those survival times a patient is both disease-free and not suffering from severe side-effects and 0 otherwise. Goldhirsch *et al.* (1989) later modified TWiST into Q-TWiST (*q*uality-adjusted TWiST) allowing the attachment of positive weights between 0 and 1 to the survival times of a patient spent in toxicity and after recurrence. The choice of suitable weights that correspond to an adequate assessment of QoL is still the subject of controversial discussion.

An intuitive approach to analyse these QAS times would be an application of standard methods of survival analysis. However, if censored observations are present, e.g. patients have not yet reached the end-point of interest at the time of analysis, as is usual in survival data, the use of individually calculated QAS can lead to serious biases in the analysis. Glasziou *et al.* (1990) have shown that transforming the natural time scale to a QAS time scale introduces informative censoring. The reason for this is that patients with low QoL will accumulate their QAS time only very slowly, therefore censoring will occur earlier in these patients than in those with a higher QoL. A proposed solution to this problem is not to calculate QAS times for the individual patient, but instead to estimate mean times of staying in a particular QoL state and then attach the QoL weights to these collective means. Unbiased treatment comparisons can be performed on the basis of these quality-adjusted mean survival times. Adjustment for the effects of additional clinical covariates by applying Cox's proportional hazards model has also been investigated for this situation (Cole *et al.*, 1993).

### Conclusions and discussion

The formal assessment of QoL of cancer patients in addition to 'hard' clinical data is becoming more and more accepted in the medical community, as reflected for example in a recent editorial in the *British Medical Journal* demanding that 'cancer trials should include measures of patients' well-being' (Byrne, 1992). The interest in QoL is demonstrated by the increasing number of publications using QoL in the title or as a keyword. In a literature survey (Schumacher *et al.*, 1991) covering *Cancer* and the *Journal of Clinical Oncology* over the 5 year period from 1985 to 1989, we found 73 such papers, of which 45 (62%) were reports of clinical trials claiming to have included QoL as an end-point of interest. However, only 36% of these QoL trials (16 out of 45) assessed QoL adequately. Eighteen per cent of the trials used insufficient measuring instruments covering only some aspects of QoL (8 out of 45); the rest used at best only surrogate end-points for QoL such as the time spent in hospital (21 out of 45). These surrogate end-points are not unimportant, but is it wrong to think that they assess QoL.

Although the above-mentioned findings are rather negative, the achievements made in some of the trials that adequately evaluated QoL have been quite remarkable. In early breast cancer, for example, several trials have investigated the efficacy of breast-preserving treatment as compared with the standard treatment of mastectomy. The results of these trials confirm that the two treatments can be regarded as equally effective with respect to survival. With regard to QoL, an overview of 18 studies conducted in this area indicates that treating breast cancer patients with breast preservation does not automatically coincide with a global improvement of their QoL as compared with the standard treatment of mastectomy (Kiebert *et al.*, 1991). This result at first seems rather surprising and counter to the often-stated contrary opinion. A possible explanation is that the negative effect that the cancer diagnosis produces on the patients' QoL overrides the effect of both surgical treatments.

Similar unexpected results were reported with respect to adjuvant treatment in cancer patients (Slevin, 1992). It is well known that chemotherapy induces toxic side-effects in a large proportion of patients, with the intensity of some of the side-effects being proportional to the intensity of treatment. However, QoL studies have found that a more intensive treatment is not always associated with lower QoL despite the objective occurrence of more severe side-effects. A possible explanation here is that the experience of going through a painful treatment provides more hope for a cure of the disease in some patients, leading to a better toleration of side-effects.

The results from these trials may serve as examples of the need to not just believe one's own assumptions about patients' QoL, but rather to let the patients assess their QoL themselves.

The successful implementation of a QoL assessment in large multicentre trials relies on the feasibility of the measurement approach. Short, self-administered, but validated questionnaires covering the major aspects of QoL should be used. The achievement of high-quality QoL data should be given the same priority as clinical data, because this is essential for adequate analysis and interpretation. Once a trial is completed, it will be impossible to improve poor data quality. Efforts to improve data quality, such as those mentioned, should be considered in the planning and implemented as part of the execution of a trial. It should be emphasised to all trial participants that the evaluation of QoL is more than just adding a new laboratory measurement. As Ganz *et al.* (1988) noted, 'it is critically important that QoL data are collected with the same care and detail as are response and toxicity data'.

In our literature survey (Schumacher *et al.*, 1991) we reviewed the statistical methods used to analyse QoL data in published trials. About one-third of the publications presented solely a descriptive analysis of the data by reporting frequencies, means and correlations. In about 50% of the trials univariate parametric or non-parametric significance tests were applied and multiple *P*-values reported. Although QoL was measured at more than two points in time in about 40% of the publications, adequate methods to analyse QoL over time were rarely used.

Although it is preferable to keep statistical methods as simple as possible to allow an easy understanding of the results, the complicated structure of QoL data often requires sophisticated analyses. Using only elementary statistical procedures in situations where they are inadequate may yield easily understandable, but also wrong, conclusions. However, as the methods for assessing QoL become more sophisticated, greater emphasis must be put into 'translating' the numerical results into understandable information for clinicians and patients to broaden the knowledge base for future treatment decisions (Cox *et al.*, 1992). For example, interpretation of the values of QoL scores calculated by an aggregation of different questions of a questionnaire may be easier when presented as the percentage of the maximum possible score instead of the raw values, and in addition makes the results more independent of the mostly arbitrary item scoring.

The proposal to combine quantity and quality of survival into one new end-point such as QAS seems intuitively appealing because end-points like total or recurrence-free survival can be regarded as special cases. It also helps to solve the problem of how to treat missing QoL data for those patients that have died. But weighting a 'hard' end-point with a 'soft' one does not create a new 'hard' end-point but rather a 'medium' one, still leaving a lot of controversy.

It is rather difficult to propose a standard statistical strategy to be adopted for an analysis of QoL data, because, unlike the situation in survival analysis, there is no typical kind of data set that calls for specific type of analysis. This is also the reason why papers concerned with the analysis of QoL data do not advocate fixed analysis plans (Schumacher *et al.*, 1991; Zee & Pater, 1991; Cox *et al.*, 1992). Because of the inherent 'softness' of QoL data it might be sensible to offer more than one adequate analysis approach, enabling interested readers to perform a sensitivity analysis for themselves.

Assessing QoL in cancer patients has reached a sound basis. Much effort has recently been put into the propagation of the idea of including QoL measures routinely in clinical research (e.g. Fitzpatrick *et al.*, 1992; Fletcher *et al.*, 1992; Slevin, 1992; Spieglehalter *et al.*, 1992; Finlay & Dunlop, 1994), but still more efforts are needed in this direction. In our view, the field of QoL evaluation requires further intensive interdisciplinary collaboration of physicians, psychosocial researchers and biostatisticians.

**References**

AARONSON, N.K., BULLINGER, M. & AHMEDZAI, S. (1988). A modular approach to quality-of-life assessment in cancer clinical trials. *Recent Results Cancer Res.*, **111**, 231-249.

AARONSON, N.K., AHMEDZA, S., BERGMAN, B. & 16 others (1993). The European Organisation for Research and Treatment of Cancer QLQ-C30: a quality-of-life instrument for use in international clinical trials in oncology. *J. Natl Cancer Inst.*, **85**, 365-376.

AGRESTI, A. (1989). A survey of models for repeated ordererd categorical response data. *Stat. Med.*, **8**, 1209-1224.

BYRNE, M. (1992). Cancer chemotherapy and quality of life: cancer trials should include measures of patients' wellbeing. *Br. Med. J.*, **304**, 1523-1524.

COLE, B.F., GELBER, R.D. & GOLDHIRSCH, A. (1993). Cox regression models for quality adjusted survival analysis. *Stat. Med.*, **12**, 975-987.

COX, D.R., FITZPATRICK, R., FLETCHER, A.E., GORE, S.M., SPIEGELHALTER, D.J. & JONES, D.R. (1992). Quality of life assessment: can we keep it simple? *J. R. Stat. Soc. Series A*, **155**, 353-392.

DE HAES, J.C.J.M., OLSCHEWSKI, M., JONAT, W., KAUFMANN, M., SCHUMACHER, M. & KOVENBAG, G. For The Zoladex Early Breast Cancer Research Association (ZEBRA) (1994). Quality of life assessment in an international, randomized trial comparing Zoladex with CMF in pre-menopausal, node-positive breast cancer patients. *Quality Life Res.*, **3**, 73-74.

DE HAES, J.C.J.M., VAN KNIPPENBERG, F.C.E. & NEIJT, J.P. (1990). Measuring psychological and physical distress in cancer patients: structure and application of the Rotterdam symptom checklist. *Br. J. Cancer*, **62**, 1034-1038.

FINLAY, I.G. & DUNLOP, R. (1994). Quality of life assessment in palliative care. *Ann. Oncol.*, **5**, 13-18.

FITZPATRICK, R., FLETCHER, A., GORE, S., JONES, D., SPIEGELHALTER, D. & COX, D. (1992). Quality of life measures in health care. I. Applications and issues in assessment. *Br. Med. J.*, **305**, 1074-1077.

FLETCHER, A., GORE, S., JONES, D., FITZPATRICK, R., SPIEGELHALTER, D. & COX, D. (1992). Quality of life measures in health care. II. Design, analysis, and interpretation. *Br. Med. J.*, **305**, 1145-1168.

GANZ, P.A., HASKELL, C.M., FIGLIN, R.A., LA SOTO, N. & SIAU, J. (1988). Estimating the quality of life in a clinical trial of patients with metastatic lung cancer using the Karnofsky Performance Status and the Functional Living Index - Cancer. *Cancer*, **61**, 849-856.

GELBER, R.D. & GOLDHIRSCH, A. (1986). A new endpoint for the measurement of adjuvant therapy in postmenopausal women with operable breast cancer. *J. Clin. Oncol.*, **4**, 1772-1779.

GLASZIOU, P.P., SIMES, R.J. & GELBER, R.D. (1990). Quality adjusted survival analysis. *Stat. Med.*, **9**, 1259-1276.

GOLDHIRSCH, A., GELBER, R.D., SIMES, R.J., GLASZIOU, P.P. & COATES, A. (1989). Costs and benefits of adjuvant therapy in breast cancer: a quality adjusted survival analysis. *J. Clin. Oncol.*, **7**, 36-44.

HÜRNY, C., BERNHARD, J., JOSS, R. & 11 others for the Swiss Group for Clinical Cancer Research (SAKK) (1992). Feasibility of quality of life assessment in a randomized phase III trial of small cell lung cancer - a lesson from the real world. *Ann. Oncol.*, **3**, 825-831.

KARNOFSKY, D.A., ABELMAN, W.H., CRAVER, L.F. & BURCHENAL, J.H. (1948). The use of the nitrogen mustards in the palliative treatment of carcinoma. *Cancer*, **1**, 634-656.

KIEBERT, G.M., DE HAES, J.C.J.M. & VAN DE VELDE, C.J.H. (1991). The impact of breast-conserving treatment and mastectomy on the quality of life of early-stage breast cancer patients: a review. *J. Clin. Oncol.*, **9**, 1059-1070.

MAGUIRE, P. & SELBY, P. (1989). Assessing quality of life in cancer patients. *Br. J. Cancer*, **60**, 437-440.

PACI, E. (1992). Assessment of validity and clinical application of an Italian version of the Rotterdam Symptom Checklist. *Quality Life Res.*, **1**, 129-134.

REGAN, J., YARNOLD, J., JONES, P.W. & COOKE, N.T. (1991). Palliation and life quality: how good are clinicians at judging treatment outcome? *Br. J. Cancer*, **64**, 396-400.

SADURA, A., PATER, J., OSOBA, D., LEVINE, M., PALMER, M. & BENNETT, K. (1992). Quality-of-life assessment: patient compliance with questionnaire completion. *J. Natl Cancer Inst.*, **84**, 1023-1026.

SCHIPPER, H., CLINCH, J., MCMURRAY, A. & LEVITT, M. (1984). Measuring the quality of life in cancer patients: the functional living index - cancer. Development and validation. *J. Clin. Oncol.*, **2**, 472-483.

SCHUMACHER, M., OLSCHEWSKI, M. & SCHULGEN, G. (1991). Assessment of quality of life in clinical trials. *Stat. Med.*, **10**, 1915-1930.

SLEVIN, M.L. (1992). Quality of life: philosophical question or clinical reality? *Br. Med. J.*, **305**, 466-469.

SLEVIN, M.L., PLANT, H., LYNCH, D., DRINKWATER, J. & GREGORY, W.M. (1988). Who should measure the quality of life, the doctor or the patient? *Br. J. Cancer*, **57**, 109-112.

SPIEGELHALTER, D.J., GORE, S.M., FITZPATRICK, R., FLETCHER, A.E., JONES, D.R. & COX, D.R. (1992). Quality of life measures in health care. III. Resource allocation. *Br. Med. J.*, **305**, 1205-1209.

TCHEKMEDYIAN, N.S. & CELLS, D.F. (eds) (1990). Quality of life in current oncology practice and research. *Oncology*, **4**, 1-232.

ZEE, B. & PATER, J. (1991). Statistical analysis of trials assessing quality of life. In *Effect of Cancer on Quality of Life*. Osoba, D. (ed.) pp. 113-124. CRC Press: Boca Raton, FL.

ZWINDERMAN, A.H. (1990). The measurement of change of quality of life in clinical trials. *Stat. Med.*, **9**, 931-942.

ZWINDERMAN, A.H. (1992). Statistical analysis of longitudinal quality of life data with missing measurements. *Quality Life Res.*, **1**, 219-224.